# Mass spectrometry imaging as a promising analytical technique for herbal medicines: an updated review

**DOI:** 10.3389/fphar.2024.1442870

**Published:** 2024-08-01

**Authors:** Jinying Zhang, Zhiguo Mao, Ding Zhang, Lin Guo, Hui Zhao, Mingsan Miao

**Affiliations:** ^1^ School of Pharmacy, Henan University of Chinese Medicine, Zhengzhou, China; ^2^ Henan Collaborative Innovation Center for Research and Development on the Whole Industry Chain of Yu-Yao, Zhengzhou, China

**Keywords:** mass spectrometry imaging, herbal medicine, spatial distribution, chemical components, mechanisms of action

## Abstract

Herbal medicines (HMs) have long played a pivotal role in preventing and treating various human diseases and have been studied widely. However, the complexities present in HM metabolites and their unclear mechanisms of action have posed significant challenges in the modernization of traditional Chinese medicine (TCM). Over the past two decades, mass spectrometry imaging (MSI) has garnered increasing attention as a robust analytical technique that enables the simultaneous execution of qualitative, quantitative, and localization analyses without complex sample pretreatment. With advances in technical solutions, MSI has been extensively applied in the field of HMs. MSI, a label-free ion imaging technique can comprehensively map the spatial distribution of HM metabolites in plant native tissues, thereby facilitating the effective quality control of HMs. Furthermore, the spatial dimension information of small molecule endogenous metabolites within animal tissues provided by MSI can also serve as a supplement to uncover pharmacological and toxicological mechanisms of HMs. In the review, we provide an overview of the three most common MSI techniques. In addition, representative applications in HM are highlighted. Finally, we discuss the current challenges and propose several potential solutions. We hope that the summary of recent findings will contribute to the application of MSI in exploring metabolites and mechanisms of action of HMs.

## Graphical Abstract

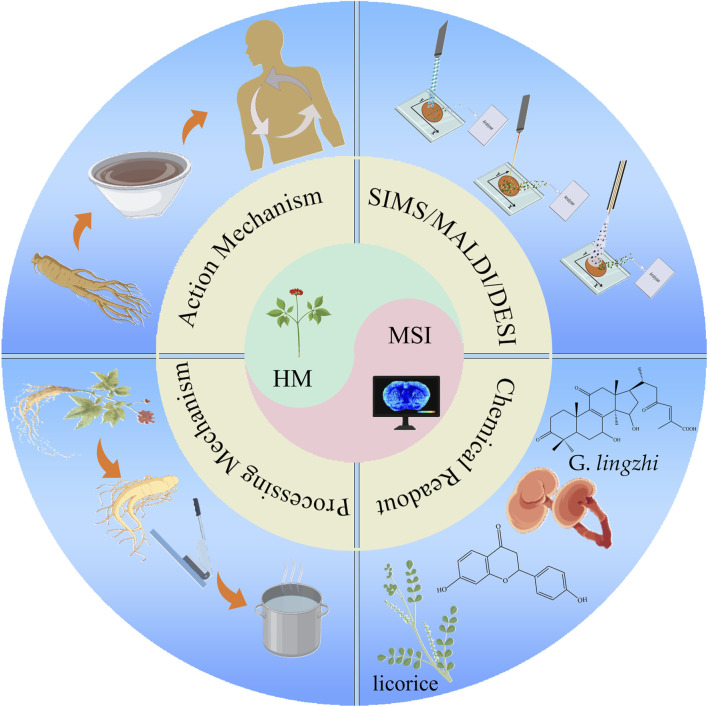

## 1 Introduction

Herbal medicines (HM), an essential component of traditional Chinese medicine (TCM), are used extensively in China and have garnered global attention for its beneficial effects and safety (Liu H. et al., 2023). Most bioactive compounds present in HMs are derived from secondary metabolites synthesized by medicinal plants, which play a key role in the therapeutic effects of HM by regulating various pathological processes within the biological system (Sturtevant et al., 2016; Gong et al., 2024). Natural herbal metabolites also serve as important resources for novel drug discovery (Hou J. J. et al., 2022). For example, artemisinin, an active antimalarial substance extracted from the botanical drug Artemisiae Annua Herba (the dried aboveground part of *Artemisia annua* L.), has highlighted the leading role of TCM in modern medicine (Tu, 2016). Nevertheless, HM is a complicated group containing diverse compounds that act simultaneously on multiple targets and pathways (Zhou et al., 2021). This inevitably leads to the uncertainty and complexity of the material basis and mechanisms of action of HM. Hence, efficient and convenient analytical approaches are required to delineate the roles of HM metabolites.

By convention, the compound analysis of HM extracts is implemented following homogenization, using common analytical methods, such as thin layer chromatography, liquid chromatography (LC), gas chromatography (GC), and mass spectrometry (MS) (Jiang H. et al., 2022). In general, LC–MS and GC–MS are two routine analytical tools allowing for qualitative and quantitative analysis of a wide range of metabolites. However, these methods often entail complex sample pretreatment (i.e., extraction, separation, and purification), which can inevitably damage unstable components. During extraction, analytes may be diluted beyond the minimum detection limit (Dong and Aharoni, 2022). More importantly, the importance of spatial distribution of metabolites in heterogeneous tissues or cells is ignored, thus hindering a comprehensive understanding of metabolites and intervention mechanisms of HMs.

Mass spectrometry imaging (MSI) has recently emerged as a powerful technique that overcomes the disadvantages of traditional analytical methods. By integrating MS analysis with ion imaging, MSI can enable precise characterization of their structural features, relative contents, and spatial distribution on the tissue surfaces (Zou et al., 2022; Ren et al., 2023). The useful technique complements traditional metabolomics and chemical analysis by integrating qualitative and quantitative molecular data with spatial information, thereby offering a more comprehensive understanding. Although the currently established molecular imaging technologies, such as computed tomography, Raman imaging, fluorescence imaging, and Magnetic Resonance Imaging, also enable *in situ* detection of metabolites (Kuzma et al., 2021), few imaging technologies can detect thousands of molecules simultaneously on a label-free basis. As a label-free ion imaging technique, MSI allows for simultaneous visualization of hundreds of analytes of interest, without prior knowledge of the metabolites present in a sample (Baijnath et al., 2022). In comparison to traditional analytical methods, MSI is characterized by high sensitivity, throughput, and spatial resolution, making it possible to spatially locate numerous endogenous and exogenous metabolites and toxic molecules without the need for solvent extraction processes (Pareek et al., 2020; Thomen et al., 2020). Therefore, MSI, as a powerful and complementary approach, has been applied extensively across scientific fields, including nutrition sciences (Yukihiro and Zaima, 2020), pharmaceutical development (Schulz et al., 2019), and clinical research (Djambazova et al., 2023; Kumar, 2023).

MSI was initially developed to visualize biomolecules (lipids, proteins/peptides, drugs, and their metabolites) in animal tissues. Given its inherent advantages, MSI has attracted considerable attention in recent years and has been expanded to the field of HMs, as first proposed by Sumner et al. at the Joint Annual Meeting of the American Fern Society (Jiang H. et al., 2022). Indeed, MSI application in botanical drugs dates back to 2007, when Wu et al. (2007a) established a matrix-assisted laser desorption ionization (MALDI) method for direct alkaloid profiling of four botanical drugs: Fuzi, processed Fuzi, Coptis chinensis Franch., and Corydalis yanhusuo W.T.Wang. Subsequently, Ng et al. (2007) applied the technique to determine the spatial distribution of secondary metabolites, especially alkaloids, in different tissue regions of *Sinomenium acutum* stem. Over the years, MSI has been increasingly used and significantly advanced in HM quality control and mechanism exploration. Without requiring tedious sample pretreatment, MSI allows for direct visualization of original compound distribution, enabling an in-depth understanding of the biosynthesis and dynamic accumulation of secondary metabolites. In animal tissues, its capacity for *in situ* analysis makes MSI well-suited for explaining complex HM mechanisms of action by characterizing tissue distributions of active metabolites or capturing endogenous biomarkers associated with HM efficacy and toxicity, enabling spatial pharmacology (Rajbhandari et al., 2024). In addition, MS-based metabolomics is a feasible method to explore mechanisms of action and find novel therapeutic targets based on the discovery of differential metabolites and prediction of differential metabolic pathways, which is consistent with the holistic view of Chinese medicine. Recently, chinmedomics strategy (i.e., the organic combination of metabolomics with serum pharmacochemistry of HM) using analytical techniques such as LC-MS and nuclear magnetic resonance has also shown great potential for discovering HM active metabolites and predicting their potential action targets, especially TCM formula (Zhang Y. et al., 2024; Wan et al., 2024). MSI-based spatial metabolomics will further promote the discovery of disease markers and multitarget exploration of HM by directly presenting the spatial position of metabolites in tissues and complementing histopathological staining and immunohistochemistry (Ren et al., 2023). MSI techniques commonly used in HMs are divided into three categories based on their ionization principles: secondary ion MS (SIMS) (Guo X. et al., 2023), MALDI (Wang J. et al., 2023), and desorption electrospray ionization (DESI) (Gao et al., 2023). A detailed comparison of the three ionization methods is presented in Table 1.

**TABLE 1:** A detailed comparison of the SIMS, MALDI, and DESI.

Ionization features	SIMS	MALDI	DESI
Introduction time	1960s	1997	2004
Ionization environment	High vacuum	High/medium vacuum	Ambient
Ionization source	High-energetic primary ion beams	Laser beam (UV/IR)	Highly charged droplets
Sample treatment	No matrix coating	Matrix coating	No matrix coating
Spatial resolution (μm)	∼0.05	5–100, lowest 1	30–200, lowest 20
Imaging scale	Cell, subcellular	Whole body, organ tissue, and cell	Whole body, organ, and tissue
Classes of analytes	Lipids, metabolites	Lipids, metabolites, glycans, peptides, proteins	lipids, metabolites, glycans, peptides
The degree of ionization	Hard	Soft	Soft
Strengths	High spatial resolution	Wide ranges of molecules	High throughput and sensitivity, easy sample pretreatment
Drawbacks	Low throughput, high degree of fragmentation, high cost	Ion suppression/matrix interferences	Low spatial resolution
Application status in TCM	Relatively rare	Very common	Increasingly common
References	Behrens et al. (2012); Heeren (2015)	Caprioli et al. (1997); Mokosch et al. (2021)	Takáts et al. (2004); Morato and Cooks (2023); Yang et al. (2023)

Several typical MSI steps, including sample preparation, data analysis, and matrix selection, have been thoroughly reviewed elsewhere (Dong et al., 2016; Yoon and Lee, 2018; Leopold et al., 2023; Ma and Fernández, 2024). Herein, we provide an overview of three MSI techniques and their key imaging principles. In addition, we mainly focus on their practical applications in visualizing HM metabolites and exploring their pharmacological and toxicological mechanisms. We especially summarize and analyze the novel application of MSI technology in elucidating processing mechanism of HMs in chemical analysis, which is a topic not covered in the previous review. Finally, current limitations and future perspectives are systematically summarized and discussed. This review aims to provide readers with a comprehensive understanding of common MSI techniques and their applications in HMs, thus facilitating further development of TCM.

## 2 Three common MSI ionization methods

### 2.1 Secondary ion mass spectrometry (SIMS)

**FIGURE 1:**
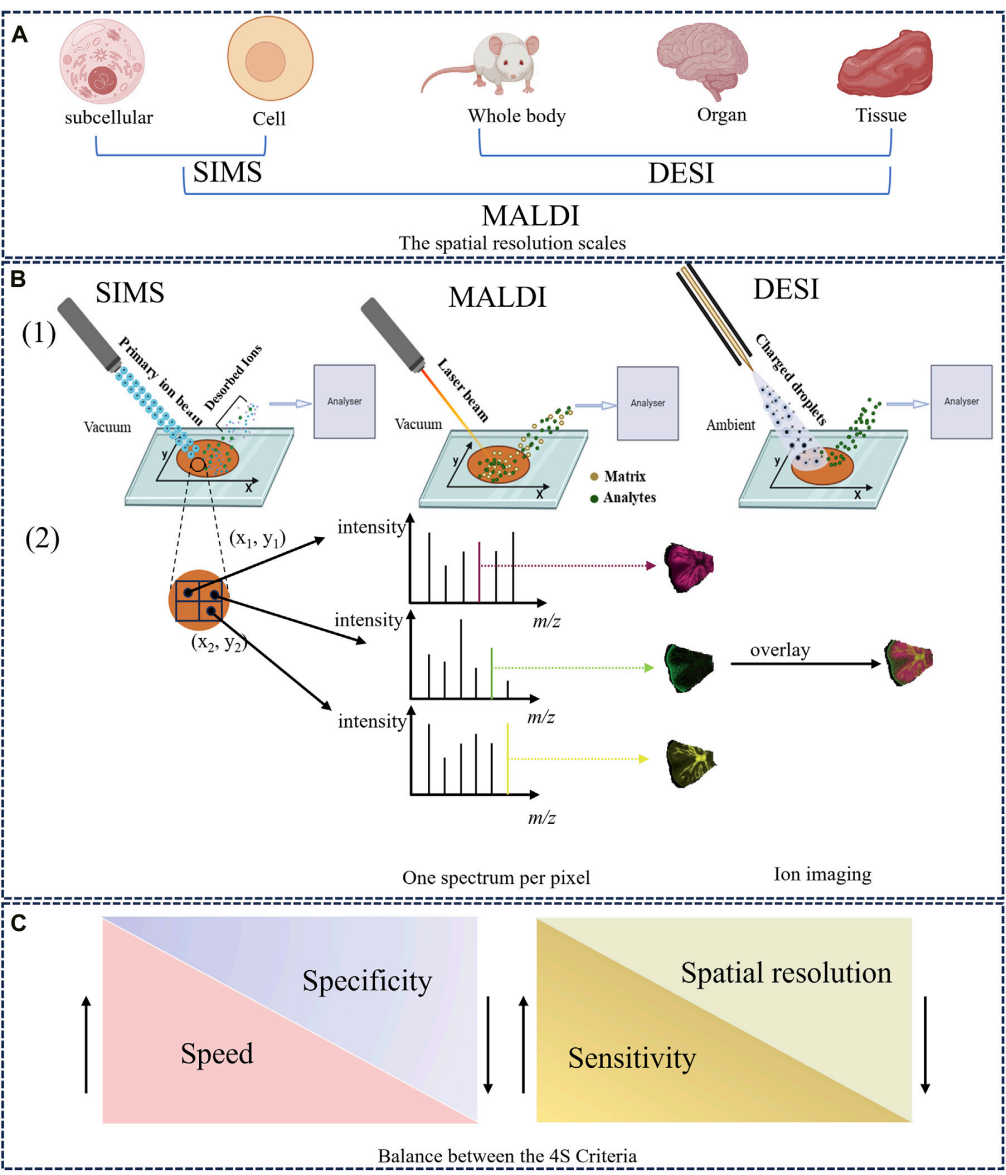
The overview of MSI technique. **(A)** The spatial resolution scales of SIMS, MALDI, and DESI. **(B)** Schematic illustration of the ionization and imaging principles of MSI. (1) A thin-section sample is placed onto a glass slide, coated with or without matrix. Ions are generated at each pixel spot by using ion beam (SIMS), a laser (MALDI) or charged solvent stream (DESI) and are measured by mass analyzer. (2) One-pixel spot corresponds to one mass spectrum. The ion intensity of each m/z peak can be extracted from individual spots and represented as a false color heat map, known as an MS image or ion image. In addition, MS images can be overlaid to compare their ion distributions (Dong and Aharoni, 2022). **(C)** The balance between the 4S Criteria (speed, specificity, spatial resolution, and sensitivity).

SIMS was first proposed by Castaing and Soldzian in the 1960s (Audinot et al., 2021), and then introduced as an imaging technique in 1983 (Bjarnholt et al., 2014). SIMS has attracted significant interest because of its extremely high spatial resolution at the sub-micrometer, and even nanometer, scale (i.e., 50–100 nm), making it especially suitable for imaging single cells and subcellular structures (Figure 1A) (Guerquin-Kern et al., 2005; Barut and Fletcher, 2023). SIMS uses a focused primary ion beam (e.g., Ga^+^, Bi^+^) with high energy (5–30 keV) to bombard the sample surface, generating a series of secondary ions. The desorbed and ionized analytes are directed into the mass analyzer for measurement (Amstalden van Hove et al., 2010; Hanrieder et al., 2013). Figure 1B provides a detailed schematic of the SIMS principle. The whole ionization process operates in a high-vacuum environment, thus the object must be freeze dried, and the process is unsuitable for unstable components (Paine et al., 2017). It is worth noting that SIMS typically images the characteristic fragments of analytes rather than their intact metabolites, owing to a very high degree of fragmentation (Vickerman, 2011). Accordingly, SIMS usually detects metabolites with low molecular weight (<1,000 Da), such as phospholipids and fatty acids (Bjarnholt et al., 2014). However, the introduction of new cluster ion beams has enabled detection of different analytes. For example, argon cluster ion beams allow for the analysis of large organic molecules such as peptides and proteins (Ninomiya et al., 2009; Lee et al., 2010). In other words, the development of gas cluster ion beams (GCIBs) with SIMS significantly decreased the fragmentation yield and improved molecular ion signals. Time-of-flight (TOF)-SIMS with GCIBs has been extensively used to investigate drug mechanisms and disease-related biomarkers in biological tissues, especially brain tissues (i.e., a highly complex, heterogeneous organ) (Sjövall et al., 2004; Bich et al., 2013; Hanrieder et al., 2013). However, the relatively high cost of SIMS compared with other imaging techniques means fewer laboratories are using it worldwide. Nonetheless, the ultra-high spatial resolution of SIMS still holds great promise for analysis of HM trace active compounds, pesticide residues, and therapeutic mechanisms.

### 2.2 Matrix-assisted laser desorption ionization (MALDI)

MALDI was first employed in 1997 to analyze biological macromolecules such as peptides and proteins (Caprioli et al., 1997). Since then, the technique has been widely applied to drug discovery (Cornett and Scholle, 2017), clinical research (Croxatto et al., 2012), and food analysis (Kokesch-Himmelreich et al., 2022). In 2002, MALDI earned its inventor the Nobel Prize in Chemistry for great contributions to biological analysis (Cho and Normile, 2002), and has emerged as the most often used and commercially available imaging device (Harkin et al., 2022). Recently, high spatial resolutions of 1.4 μm and 1 μm have been achieved with MALDI–MSI (Kompauer et al., 2017) and atmospheric pressure-scanning microprobe MALDI–MSI (Mokosch et al., 2021), respectively.

Several steps are fundamental to the desorption/ionization mechanisms of MALDI. First, the appropriate matrix material is evenly sprayed on the surface and co-crystallized with analytes on the metal plate, generating matrix-analyte complexes (Wang J. et al., 2023). Next, a pulsed ultraviolet or infrared laser beam bombards the sample surface; its energy is directly absorbed by the matrix, and then transferred to the analyte molecules. Finally, the matrix is vaporized, carrying intact sample molecules into the gas phase, leading to their desorption and ionization (Bjarnholt et al., 2014). A classic MALDI ionization process is described in Figure 1B. During the process, some ions (e.g., H^+^, Na^+^) can be exchanged between the matrix and analyte, producing numerous quasi-molecular ions (Engel et al., 2022). Accordingly, MALDI is a relatively soft ionization method, generating singly charged ions with a wide mass/charge number (m/z) range (Koestler et al., 2008). Large biomolecules in biological samples (e.g., proteins, lipids, peptides) are typically imaged by MALDI–MSI (Spengler, 2015).

In MALDI–MSI analysis, the selection and optimization of a matrix plays a pivotal role in ion imaging. It both facilitates ionization of analytes that do not absorb light energy themselves and affects spatial resolution by impacting the displacement of analytes on the sample surface (Vallianatou et al., 2019; Guo X. et al., 2023). However, a matrix effect exists during imaging, suppressing or enhancing analyte ionization efficiency based on other analytes present in the sample or biological matrix (Rajbhandari et al., 2024). A high matrix ion signal can lead to interference at the low mass range, significantly affecting detection of small molecules (m/z < 750 Da) (Guo X. et al., 2023). Hence, an ideal matrix material is characterized by the smallest possible crystal size and a low ion signal background. Common matrices are organic acid compounds, like 2,5-dihydroxybenzoicacid, sinapic acid, and α-cyano-4-hydroxycinnamicacid. A list of matrices for this technique, and corresponding analytes, are in Table 2. As more classes of novel matrices with high analyte desorption/ionization are discovered and applied [e.g., nitro indole derivatives (Liang et al., 2024), 2-hydrazinoterephthalic acid (Wang H. et al., 2023), 3,4-dimethoxycinnamic acid (He et al., 2019)], MALDI will become more applicable for analyzing diverse compounds. This issue has been elaborated on previously (Engel et al., 2022; Jiang H. et al., 2022; Guo X. et al., 2023). In addition, like SIMS, MALDI is typically carried out under high vacuum conditions, necessitating relatively complicated sample preparation compared with ambient conditions. Freezing microtome sectioning is essential for biological tissues, which are easily deformed in the vacuum, destroying information about their original spatial distributions (Zhu et al., 2021). Consequently, there is a growing demand for ionization techniques that do not rely on a matrix coating and can operate in real-world environments.

**TABLE 2:** Commonly used matrixes for MALDI-MSI.

Matrixes	Analytes	Reference
2,5-Dihydroxybenzoicacid	Lipids, amino acids, and oligosaccharides	Bjarnholt et al. (2014); McMillen et al. (2020)
1,5-Diaminonaphthalene	Phospholipids, fatty acids, amino acids and nucleotides	Korte and Lee (2014); Jiang H. et al. (2022)
9-Aminoacridine	Amino acids, nucleotide derivatives, and lipids	Morikawa-Ichinose et al. (2019)
α-Cyano-4-hydroxycinnamicacid	Peptides, lipids, and glycoproteins	Laugesen and Roepstorff (2003); Jiang H. et al. (2022)
3-Hydroxypicolinic acid	Oligonucleotides	Yokoi et al. (2018)
Sinapic acid	Large proteins, oligosaccharides, and glycoproteins	Botting (2003); Guo X. et al. (2023)
2,5-Dihydroxyacetophenone (DHAP)	Large proteins, peptides, and glycoproteins	Wenzel et al. (2006); Jacksén and Emmer (2012)

### 2.3 Desorption electrospray ionization (DESI)

Based on MALDI, Cooks et al. (Wu et al., 2013) initially introduced DESI in 2004 and used this novel ionization source to image biological tissues in 2006 (Wiseman et al., 2006), initiating a new field of ionization at atmospheric pressure. That DESI can be used directly in ambient environments, without vacuum requirements, which is the vital feature distinguishing it from MALDI and SIMS (Takáts et al., 2005). This technique is widely popular because it requires fewer complex sample treatments and no matrix compound, thus also avoiding matrix interference. The ionization mechanism for DESI was described by Cooks et al. as a “droplet pick-up” process (Morato and Cooks, 2023).

In this method, a charged solvent spray is first generated by a constant flow rate (μL/min) of solvent (e.g., methanol, acetonitrile, dimethylformamide) under a high voltage potential and a nebulizer gas (typically nitrogen). When the charged spray micro-droplets are directed toward a sample surface, sample molecules (mostly metabolites and lipids in animal tissues) are extracted, desorbed, and ionized, producing a series of gaseous ions (Talaty et al., 2005). Finally, desorbed ions enter the mass analyzer through an ion transfer line. A simple schematic of this process is provided in Figure 1B. DESI, a soft ionization process, allows the analysis of intact polar molecules with low molecular weight (Takáts et al., 2004; Kafeenah et al., 2022). Nevertheless, since the solvents used are usually hydrophilic, DESI is inappropriate for non-polar metabolites and its sensitivity to proteins and other large biomolecules is not also ideal. Compared with SIMS and MALDI, DESI possesses a lower spatial resolution (180–220 μm) because the spray area cannot be accurately controlled (Ifa et al., 2010). Recently, the introduction of nano-DESI has enhanced the spatial resolution imaging to 40 μm (Mesa Sanchez et al., 2022). This lower spatial resolution can be compensated for by high throughput and sensitivity according to the 4S-criteria (Figure 1C). Working in air, DESI can be used with various sample types, including fresh-cut plant tissues, animal tissues, and biological liquids (e.g., urine, plasma) (Takáts et al., 2005). With its advantages of high throughput and easy sample preparation, DESI–MSI is increasingly applied in HM research.

### 2.4 The key imaging principles

As a well-established molecular imaging technique, MSI involves different ion source types coupled to mass analyzers (e.g., TOF, ion trap analyzers), which are largely responsible for the mass accuracy, mass resolution, and quality of ion imaging (Guo X. et al., 2023). Differences among MSI techniques mainly depend on how the analytes are ionized. In the MSI process, a large number of metabolites from every pixel point on the sample are first desorbed and ionized by an energetic primary ion beam, laser beam, or charged microdroplets, all of which generate ions (Dong and Aharoni, 2022). The produced ions enter the mass analyzer and are separated according to their m/z, and detected. Finally, the spatial positions of analytes are recorded within the original imaging dataset by mapping an x-y array of coordinates along with their relative abundance. In general, one pixel corresponds to a mass spectrum, thus a large number of mass spectra can be obtained for a tissue slice (Figure 1B) (Schulz et al., 2019). During data analysis, MSI software enables the extraction of the ion intensity value of a targeted m/z peak within the specific mass spectrum and plots these using different color scales, thus constructing an MS image that represents the distribution of a compound of interest (Bjarnholt et al., 2014). More importantly, the spatial distributions of several ions in a sample can be compared by overlaying their MS images, as shown in Figure 1B.

When using MSI, four important parameters (spatial resolution, speed, sensitivity, and specificity), which mutually constrain each other, are subject to the 4S-criteria (Figure 1C) (Schulz et al., 2019). For example, when high spatial resolution is the experimental priority, sensitivity and throughput decreases are inevitable due to the increased pixels and decreased sampling volume. Consequently, selecting the appropriate integration between ionization source and mass analyzer (e.g., MALDI–TOF, SIMS–TOF, MALDI–Orbitrap) is crucial for successfully detecting and imaging the desired analytes, which depends on the sample dimensions and properties. For instance, when imaging differential metabolites at the cellular level, MALDI should be prioritized for its high spatial resolution and wide mass range. For mass analyzer, TOF would be preferred for its high mass resolution and accuracy especially when combined with MALDI (i.e., MALDI–TOF).

Herein, we have briefly discussed the three most common MSI techniques and their ionization mechanisms. More detailed explanations and introductions have been presented in several excellent reviews (Cooks et al., 2006; Fuchs et al., 2010; Guo X. et al., 2023; Jia et al., 2023).

## 3 MSI application in chemical analysis of HM

Secondary metabolites (e.g., saponins, flavonoids, alkaloids) in medicinal plants are not only involved in defensive functions and signal transmissions but serve as the material basis for HM efficacies. However, the distribution of these metabolites in HM is usually heterogeneous. MSI makes it possible to visualize the spatial distribution of functional HM compounds, allowing direct indications for an in-depth understanding of their biosynthetic processes and revealing where and how they are enriched. Moreover, visualizing the spatial distribution of metabolites in both crude and processed HMs facilitates the identification of quality markers and uncovers potential chemical transformation mechanisms during the processing procedure, known as Pao Zhi in Chinese. This approach not only aids in refining processing methodologies but also ensures the safety of medications, thereby enhancing overall product quality and efficacy.

### 3.1 Direct readout of spatial location of medicinal compound

The spatial distribution of compounds within HMs varied across medicinal tissues. However, traditional analytical methods can only identify and analyze the metabolites, failing to provide specific spatial information. As a complement to traditional chemical analysis, MSI allows non-labeled *in situ* metabolite analysis, and enables their precise location. Furthermore, the types and contents of active metabolites in HMs are also affected by factors such as growth stage, medicinal portion, cultivation year, microregion, and growing environment. MSI directly reveals overall differences in metabolites and abundance by combining chemometric analysis. By showing spatial distribution of chemical components within heterogeneous HM tissues, MSI can provide researchers with more contextualized information. The MSI methods, research drugs, sample types, and imaged analytes involved in this article are summarized in Table 3, categorized according to research objectives and chronology.

**TABLE 3:** A summary of MSI applications in metabolite analysis and mechanisms of action of HMs.

Purposes	Medicinal plants or drugs	Tissue types	Imaging technique	Imaged analytes	References
Metabolite distribution (medicinal plants)	*Periplaneta americana* (Family: Blattidae, Genus: *Periplaneta*)	Whole body	MALDI-MSI	N-acetyldopamine oligomers	Zhang J. et al. (2024)
	Ganoderma lingzhi (Ganodermataceae; Ganoderma lucidum or Ganoderma sinense)	Dried fruiting body	DESI-MSI	Metabolites	Xia et al. (2024)
	*Scutellaria baicalensis* (Family: Lamiaceae; Genus: *Scutellaria*)	Whole body	MALDI-MSI	Metabolites	Zhou et al. (2024)
	Fuzi (Ranunculaceae; *Aconitum carmichaelii* Debeaux)	Lateral roots	DESI-MSI	Paclobutrazol	Hou et al. (2023)
	Angelica pubescens (Family: Umbelliferae; Genus: *Angelica*)	Root	MALDI-MSI	Coumarins	Li et al. (2023c)
	Rauvolfia tetraphylla (Family: Apocynaceae; Genus: *Rauvolfia*)	Stem, roots, and leaves	MALDI and DESI-MSI	Monoterpenoid indole alkaloids	Lorensen et al. (2023a)
	Puerariae lobata and Puerariae thomsonii (Family: Leguminosae; Genus: *Puerariae*)	Root	DESI-MSI	Metabolites	Guo N. et al. (2023)
	Salvia miltiorrhiza Bunge (Lamiaceae; Salviae miltiorrhizae radix et rhizoma)	Root and rhizome	DESI–MSI	Diterpenoids	Xia et al. (2023)
	Isatidis Radix (*Brassicaceae*; Isatis indigotica)	Root	MALDI and DESI-MSI	Quality markers	Nie et al. (2023)
	Angelica sinensis (Umbelliferae; Angelica sinensis (Oliv.) Diels)	Root	MALDI-MSI	Volatile oil	Li Q. et al. (2023)
	Chinese jujube (*Rhamnaceae*; Ziziphus jujuba Mill.)	Fruit	MALDI-MSI	Metabolites	Lu D. et al. (2023)
	Isatidis radix	Root	DESI-MSI	Metabolites	Nie et al. (2022)
	Forsythia suspensa (Oleaceae; Forsythia suspensa (Thunb.) Vahl)	Fruit	MALDI-MSI	Metabolites	Jing et al. (2022)
	*Gelsemium elegans* (Family: Gelsemicaeae; Genus: *Gelsemium*)	Roots, stems, and leaves	DESI-MSI	Alkaloids	Wu et al. (2022a)
	Salvia miltiorrhiza Bunge	Whole body	DESI-MSI	Metabolites	Tong et al. (2022)
	Cordyceps sinensis (Clavicipitaceae; cordyceps sinensis (Berk.) Sacc)	Whole body	TOF-SIMS	Active metabolites	Liu Q. B. et al. (2022)
	Coptis chinensis (Ranunculaceae; Coptis chinensis Franch., Coptis deltoidea C.Y.Cheng et Hsiao., and Coptis teeta Wall.)	Rhizome	TOF-SIMS	Alkaloids	He et al. (2022)
	Cordyceps sinensis	Whole body	TOF-SIMS	Active metabolites	(M.-C. et al., 2022)
	*Arctium lappa* L. (Family: Asteraceae; Genus: *Arctium*)	Root	MALDI-MSI	Metabolites	Li et al. (2022)
	Paris polyphylla var. yunnanensis (Family: Veratriceae; Genus: *Paris*)	Rhizome	MALDI-MSI	Metabolites	Zhang G. H. et al. (2022)
	Coptis chinensis	Rhizome	DESI-MSI	Metabolites	Wang et al. (2022)
	*Paeonia lactiflora*	Root	DESI-MSI	Active metabolites	Chen et al. (2022)
	*Paeonia suffruticosa* and *Paeonia lactiflora* (Family: *Paeoniaceae*)	Root	MALDI-MSI	Metabolites	Li B. et al. (2021)
	Lotus Seed (Nymphaeaceae; *Nelumbo nucifera* Gaertn.)	Seeds	MALDI-MSI	Metabolites	Sun C. L. et al. (2021)
	Wolfberry fruit (Solanaceae; *Lycium barbarum* L.)	Fruit	MALDI-MSI	Metabolites	Zhao et al. (2021)
	Heshouwu (*Polygonaceae*; Polygonum multiflorum Thunb)	Root tubers	MALDI-MSI	Related markers	Wiseman et al. (2006)
	*Salvia miltiorrhiza* Bunge	Root and rhizome	MALDI-MSI	Metabolites	Sun et al. (2020a)
	*Scutellaria baicalensis* (Family: Lamiaceae; Genus: *Scutellaria*)	Root	MALDI-MSI	Flavones	Sun et al. (2020b)
	*Ginkgo biloba* L. (Family: *Ginkgoaceae*; Genus: *Ginkgo*)	Leaves	MALDI-MSI	Metabolites	Li et al. (2018)
	Psychotria prunifolia (Family: *Rubiaceae*; Genus: *Psychotria*)	Leaves	DESI-MSI	Alkaloids	Kato et al. (2018)
	*Paeonia lactiflora*	Root	MALDI-MSI	Active components	Li B. et al. (2016)
	*Ginkgo biloba* L.	Leaves	MALDI-MSI	Flavonoid glycosides and bioflavonoids	Beck and Stengel (2016)
	*Panax ginseng* (*Araliaceae*; *Panax ginseng* C.A.Mey.)	Root	MALDI-MSI	Ginsenosides	Taira et al. (2010)
	Fuzi	lateral root	MALDI-MSI	Alkaloids	Wang et al. (2009)
Processing mechanisms (medicinal plants)	Fuzi and Processed Fuzi	Lateral root	MALDI-MSI	Alkaloids	Wu et al. (2007a)
	*Panax quinquefolius* processed product (Araliaceae; *Panax quinquefolium* L.)	Root	MALDI-MSI	Ginsenosides	Li H. Z. et al. (2023)
	Morindae Officinalis Radixp processed products (*Rubiaceae*; Morinda officinalis How)	Root	MALDI-MSI	Iridoid and saccharousins	Qiao et al. (2022)
	Aconiti Radix Cocta processed product (Ranunculaceae; *Aconitum carmichaelii* Debx.)	Mother root	MALDI-MSI	Alkaloids	Dai et al. (2022)
	Gastrodiae Rhizoma processed product (*Orchidaceae*; Gastrodia elata Bl.)	Tuber	MALDI-MSI	Phenols	Harkin et al. (2022)
	Ligustri Lucidi Fructus and wine-processed product (*Oleaceae*, Ligustrum lucidum Ait.)	Fruit	MALDI-MSI	Related markers	Li et al. (2020)
	*Panax notoginseng* processed product (*Araliaceae*; *Panax notoginseng* (Burkill) F.H.Chen)	Root and rhizome	MALDI-MSI	Active components	Sun C. et al. (2021)
	Strychnos nux-vomica seeds and its processed products (Loganiaceae; Strychnos nux-vomica L.)	Seeds	MALDI-MSI	Alkaloids	Wu et al. (2007b)
	Aconitum pendulum and its processed product (Family: *Polygonaceae*; Genus: *Aconitum*)	Root	DESI-MSI	Differential metabolites	Tan et al. (2023)
	Raw and processed fuzi	Lateral root	DESI-MSI	Aconitum alkaloids	Liu Y. et al. (2022)
	Raw and processed *Panax notoginseng*	Root	MALDI-MSI	Ginsenosides	Fan et al. (2022)
	Bombyx batryticatus and stir-fried product (*Bombycidae*; *Bombyx mori* Linnaeus)	Whole body	MALDI-MSI	Protein-related metabolites	Liu P. et al. (2023)
Metabolic distribution (animal tissues)	*Uncaria rhynchophylla* (Miq.) Miq. ex Havil. (*Rubiaceae*; *Rubiaceae*)	Rat brain	DESI-MSI	Uncaria alkaloids	Gao et al. (2022)
	Xiaoke pills	Zebrafish	DESI-MSI	Chemical components and their metabolites	Zhu et al. (2022)
	*Rubia tinctorum* (Family: *Rubiaceae*; Genus: *Rubia*)	Rat kidney	DESI-MSI	Anthraquinone	Ishii et al. (2022)
	Scutellarin	Mouse kidney	MALDI-MSI	Scutellarin and metabolites	Wang T. et al. (2021)
	Ginsenoside Rg1	Rat organs	DESI-MSI	Ginsenoside Rg1	Wei et al. (2021)
	Salidroside	Mouse organs	MALDI-MSI	Scutellarin and scutellarein	Meng et al. (2020)
	Paclitaxel	Mouse body	Virtual calibration quantitative MSI	Paclitaxel and its prodrug	Zhang et al. (2020)
	*Stephania tetrandra* S. Moore. (Family: *Menispermaceae*; Genus: *Stephania*)	Rat organs	MALDI–MSI	Tetrandrine	Tang et al. (2019)
	*Ephedra sinica* (Family: *Ephedraceae*; Genus: *Ephedra*)	Rat lung	MALDI-MSI	Ephedrine	Matsumoto et al. (2017)
	Puerarin	Mouse kidney	GD-4-assisted MSI	Puerarin and its two metabolites	Shi et al. (2017)
	Vinblastine	Rat whole body	MALDI–MSI	Vinblastine and metabolites	Trim et al. (2008)
Pharmacological and toxicological mechanisms (animal tissues)	heshouwu	Mouse liver	DESI-MSI	Endogenous metabolites	Jiang H. Y. et al. (2022)
	Aristolochic acids	Rat kidney	DESI-MSI	Endogenous metabolites	Wang et al. (2020)
	Pterostilbene	Rat brain	DESI-MSI	Endogenous metabolites	Ban et al. (2024)
	Ningxin fomula	Rat heart	MALDI-MSI	Endogenous metabolites	Huang et al. (2023)
	Yi-Xin-Shu capsule	Rat heart	MALDI-MSI	Endogenous metabolites	Zhang M. et al. (2022)
	Ginseng and *Panax quinquefolius*	Rat brain	DESI-MSI	Endogenous metabolites	Huang et al. (2023)
	Fritillariae cirrhosae bulbus (Liliaceae; *Fritillaria taipaiensis* P. Y. Li)	Rat lung	MALDI-MSI	Endogenous metabolites	Qin et al. (2024)
	Notoginseng leaf triterpenes	Rat brain	MALDI–MSI	Endogenous metabolites	Wang L. et al. (2021)
	XueFu ZhuYu decoction	Rat brain	nanoDESI-MSI	Endogenous metabolites	Li Y. et al. (2021)
	Notoginsenoside R1	Rat brain	MALDI–MSI	Endogenous metabolites	Zhu et al. (2020)
	Thymoquinone	Rat brain	MALDI–MSI	Endogenous metabolites	Tian et al. (2020)
	Fuzi	Rat heart	MALDI-MSI	Endogenous metabolites	Wu et al. (2019b)
	Shenfu injection	Rat heart	MALDI-MSI	Endogenous metabolites	Wu et al. (2019a)
	N(6)-(4-hydroxybenzyl)-adenosine	Rat whole body	DESI-MSI	Endogenous metabolites	He et al. (2015)

**FIGURE 2:**
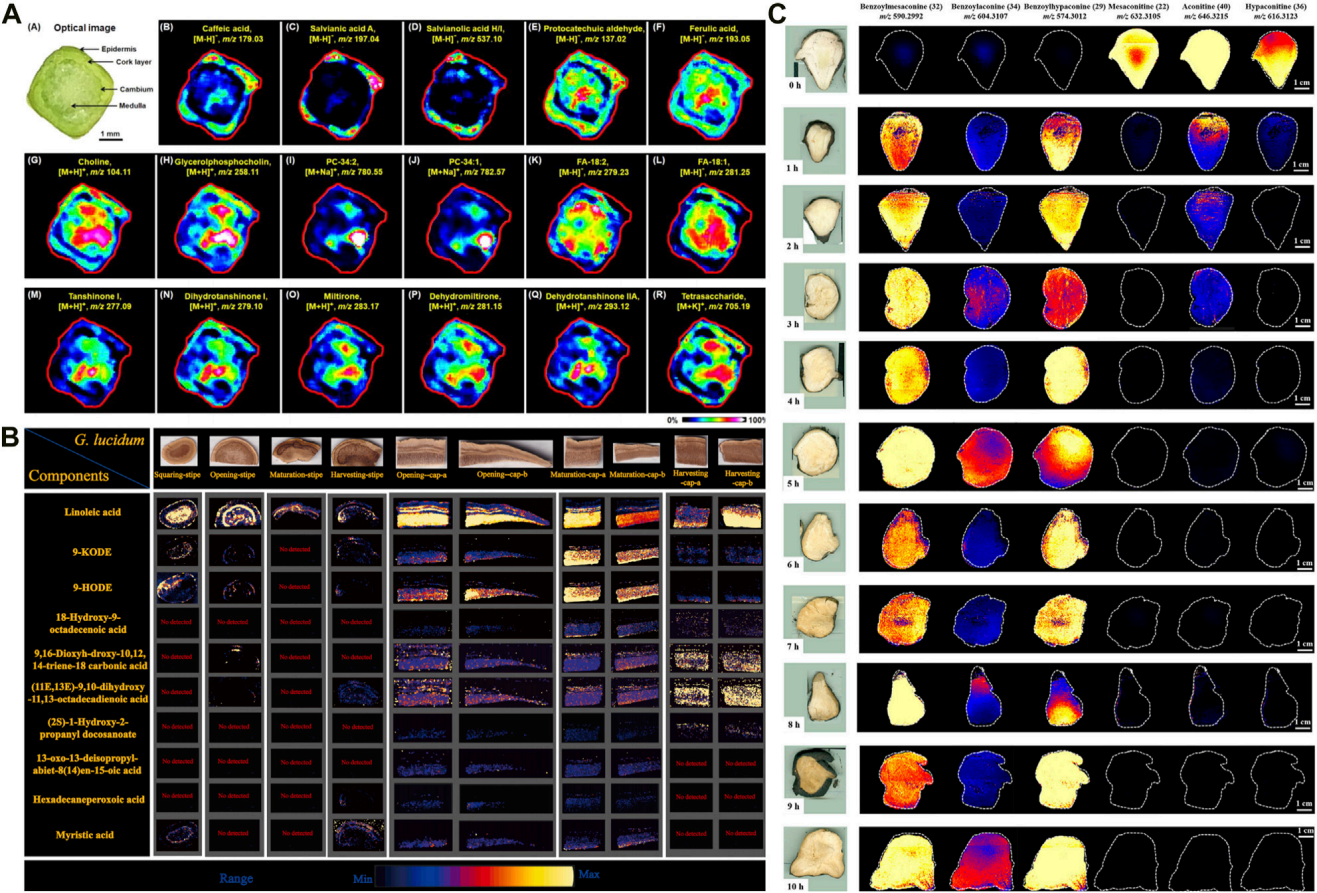
Application of mass spectrometry imaging in chemical analysis of herbal medicines. **(A)** The distribution of representative metabolites in *Salvia miltiorrhiza Bge* stem microregions (Sun et al., 2020a). **(B)** DESI-MSI spatial distribution of the ganoderic acids in different tissues of *G. lingzhi* at different maturity stages (Xia et al., 2024). **(C)** DESI-MS images of six ester-type alkaloids in raw and processed Fuzi steamed for different time points (Liu Y. et al., 2022).

In the HM context, SIMS has been used to image several medicinal plants to determine the spatial distributions of specific trace substances, such as *Cordyceps sinensis* (e.g., mannitol, cordycepin, ergosterol) (Li Z. P. et al., 2016; Xia et al., 2021; Meng-Chan et al., 2022) and *Coptis chinensis rhizome* (e.g., berberine, epiberberine, coptisine) (He et al., 2022). These findings have provided the valuable reference for HM quality control. For example, TOF-SIMS was used to analyze the distribution of compounds in the cross-section of *C. chinensis rhizome*. Palmatine was found to be more widely distributed in the pith compared to other parts, while oxyberberine was primarily concentrated in the cork and xylem rays. Meanwhile, the findings were further verified using HPLC coupled with triple quadrupole MS. In addition, Sun et al. (2020a) developed a high-coverage MALDI–MSI method, primarily to profile the spatial locations and dynamic changes of a wide range of endogenous small molecules, including amino acids, phenolic acids, and fatty acids in diverse structures (e.g., cork, cortex, phloem, cambium, xylem, medulla) of the root and stem of *Salvia miltiorrhiza Bunge*[Lamiaceae; Salviae miltiorrhizae radix et rhizoma]. Interestingly, tanshinone metabolite—which is traditionally obtained from the root—was also observed in the stem (Figure 2A). The result can contribute to the further selection of medicinal parts of *S. miltiorrhiza Bge*. Subsequently, another study used DESI–MSI to characterize and visualize diverse metabolites in different parts of the medicinal plant (e.g., roots, stems, leaves, and flowers), and to visualize the biosynthesis of flavonoids and phenolic acids (Tong et al., 2022). The finding revealed that danshensu was identified as a key precursor for the generation of phenolic acids in *Salvia* species. In another study, DESI–MSI and multiomics analysis unveiled the spatial distribution and biosynthesis mechanisms of diterpenoid tanshinones and polyphenolic salvianolic acids in the root and leaves of *S. miltiorrhiza Bunge*, respectively, and determined the key genes expressed in this pathway (Xia et al., 2023). These findings enhance our understanding of the precise locations and biosynthesis of metabolites, especially of the effective compounds in *S. miltiorrhiza*. In exploring the root of *Paeonia lactiflora* (PL), atmospheric pressure-scanning microprobe MALDI–MSI was performed to visualize cellular-level distributions of gallotannins, monoterpene glucosides, and other secondary metabolites (Li B. et al., 2016). The study revealed that gallotannins were primarily accumulated in the cork and xylem regions, where they serve a defensive function against external damage. The relationship between physiological function and spatial distribution has been established. Nevertheless, many isomers including the two most important active secondary metabolites: paeoniflorin and albiflorin could not be well distinguished using MSI technique alone. In another comparative study, ion images of major intermediates participating in the biosynthetic pathway of gallotannins were achieved in the root sections of *P. suffruticosa* (PS) and PL. More importantly, researchers developed tandem MSI to eliminate ambiguity in the spatial distribution of the isomers paeoniflorin and albiflorin. The results showed that these bioactive constituents were also accumulated in the corks of PS and PL roots, indicating the desirability of preservation of the cork layer as much as possible in the production and processing of crude drugs (Li B. et al., 2021; Chen et al., 2022).

Ginsenosides are important active compounds, present extensively in the family Araliaceae (e.g., *Panax quinquefolius*, Ginseng, and *P. notoginseng*). A growing number of studies have identified and imaged these using MSI. Recently, DESI–MSI and UPLC-Q-TOF/MS were used jointly to identify 15 differential metabolites in different microregions of *P. quinquefolius* [Araliaceae; *P. quinquefolium* L.]. Among them, ginsenosides were the main differential biomarkers distinguishing the outer core from the center. Additionally, higher levels of ginsenosides, acting as defensive plant regulators, were detected in the cork and phloem compared with the medulla and xylem (Taira et al., 2010; Luo et al., 2024). This study demonstrated tissue-specific distributions of metabolites and revealed a direct connection between this distribution and the physiological functions of the metabolites. Likewise, MALDI was used to comprehensively describe the spatial distributions of metabolites in *P. notoginseng* [Araliaceae; *P. notoginseng* (Burkill) F.H.Chen] (Sun C. et al., 2021). The differentially expressed ginsenosides in Ginseng [Araliaceae; *Panax ginseng* C.A.Mey.] and *P. notoginseng* across cultivation years were visualized using the same method, revealing quality differences (Liu et al., 2020; Yang et al., 2021; Lu Y. et al., 2023). In another ginseng species [Zhu-Zi-Shen, [Araliaceae; *Panax japonicus var. major* et rhizome)], DESI–MSI showed that ginsenosides accumulated predominantly in the cork and phloem, and a large number of unknown structures were unveiled primarily. These studies provide valuable insights into the accurate locations within targeted tissues, accumulation patterns, and tissue-specific extraction of saponins. However, differentiating among many isomeric ginsenosides remains challenging due to the limited analytical capabilities of DESI (Jiang M. et al., 2023).

Licorice [Fabaceae; *Glycyrrhiza uralensis* Fisch. et rhizome] is a common medicinal plant with a homology of medicine and food. Functional small molecule metabolites (i.e., flavonoids and saponins) present in licorice have been characterized in various microregions by MALDI–MSI and DESI–MSI. These results suggest that flavonoids are primarily accumulated in the cork, phloem, and medulla of the rhizome, while triterpenoids are widely distributed in the medullary, xylem, and phloem near the cork layer (Li et al., 2014). Similarly, MSI cannot separate or precisely identify isomers without tandem MS, which leads to the loss of chemical information in licorice. Thus, complementary methods are required for analyzing compounds with the same or similar molecular weights. In a recent study regarding licorice, the ion mobility (IM) technique was employed to add an extra isolation dimension to MSI. Researchers used DESI–MSI to detect 21 flavonoids and 12 triterpenoids in different areas of rhizome tissue. In subsequent work, the research team integrated DESI–MSI and HILIC/IM-QTOF-HDMS^E^ (hydrophilic interaction chromatography/ion mobility-quadrupole time-off light high-definition) to directly characterized the distribution of oligosaccharides with eight different degrees of polymerization (Zhao et al., 2023), offering more comprehensive information for sustainable utilization and further development licorice. DESI–IM–Q/TOF was also applied to spatially visualize the seeds of peach, bitter almond, and Chinese dwarf cherry, observing that most unique compounds, especially amygdalin, were more abundant at the edges of the seed kernels than in the centers of the cotyledons (Hou J. et al., 2022). The finding provided us a hint that long-term soaking of bitter almond seeds should be avoided, to prevent amygdalin hydrolysis. Additionally, another important class of active components, flavonoids, were also fully mapped in HMs such as Ginkgo biloba L., Cannabis sativa, and Scutellaria baicalensis Georgi (Beck and Stengel, 2016; Li et al., 2018; Sun et al., 2020b; Lorensen et al., 2023b). Such studies can further enhance our understanding of the biosynthesis and biological functions of secondary HM metabolites.

Metabolite accumulation patterns and abundances are dynamic across growth stages and tissue parts. MSI can be used to investigate these aspects by visualizing their heterogeneous distribution, which can help determine optimal harvest periods and advance rational HM use. For example, *Ganoderma lingzhi* (*G. lingzhi*) [Ganodermataceae; Dried fruiting body of Ganoderma lucidum or Ganoderma sinense] is valued for its high medicinal and nutritional value. Xia et al. developed DESI–MSI combined with LC–MS plant metabolomics to explore the dynamic accumulation patterns of *G. lingzhi* components during four maturity stages (i.e., squaring, opening, maturation, and harvesting). Approximately 132 metabolites, including 115 triterpenoids, 11 fatty acids, and other components, were identified. Further analysis revealed a higher concentration of ganoderic acids in caps compared with stipes. The abundance of most ganoderic acids decreased in both stipe and cap as *G. lingzhi* matured. The accumulation of fatty acids was predominantly observed during the opening and maturing stages of the caps (Xia et al., 2024) (Figure 2B). These findings provide a basis for the rational use of medicinal parts of *G. lingzhi*., and trace how its triterpenes accumulate. Over recent years, parallel studies of other medicinal plants have been conducted, including Jujube (Lu D. et al., 2023), Forsythia suspensa (Jing et al., 2022), *Gelsemium elegans* (Wu et al., 2022a; Wu et al., 2022b), and wolfberry fruit at different development stages (Zhao et al., 2021).

In TCM, *Angelica sinensis* [Umbelliferae; *A. sinensis* (Oliv.) *Diels* et root] are routinely divided into the head, body, and tail according to their distinct clinical medicinal properties. Traditionally, the body is used to replenish blood, while the head and tail promote blood circulation (Yang et al., 2017). However, the material basis for these differential effects had not been fully elucidated. Therefore, both fluorescence imaging and MALDI–MSI were used to perform a comparative analysis of the root’s volatile oil. Fluorescence imaging suggested significant differences in the content of the total volatile oil between the head, body, and tail. Meanwhile, the specific spatial distribution of each compound was also revealed by MSI (Li Q. et al., 2023). This offers a novel approach to understanding the pharmacological differences across medicinal parts of *A. sinensis*. MSI-based spatial metabolomics combined with multivariate statistical analysis (principal component analysis, PCA; orthogonal partial least squares discriminant analysis, OPLS-DA) were used to discriminate HM adulteration (Zhao et al., 2021) and other quality characteristics (Nie et al., 2022; Nie et al., 2023).

In addition to the abovementioned medicinal plants, the use of MSI has expanded to spatially resolved analysis of active constituents related to animal-based TCMs. For instance, *Periplaneta americana* [Family: Blattidae, Genus: Periplaneta] is a medicinal insect that improves blood circulation and alleviates blood stasis. Fifteen N-acetyldopamine oligomers with anti-inflammatory and vasorelaxant effects were extracted from the insect, and their distribution throughout the insect was visualized using MALDI–MSI. This analysis indicated that N-acetyldopamine oligomers were more highly distributed in peripheral parts (Zhang J. et al., 2024). That study presented an innovative approach to examining active substances in animal-derived medicines. Collectively, MSI application to the chemical analysis of HM provides direct guidance for their quality control.

### 3.2 Chemical perspectives on processing (Pao Zhi) mechanisms of HM

Processing (Pao Zhi) is a unique Chinese pharmaceutical technique for attenuating toxicity and altering HM efficacy (Pan et al., 2023). In general, most HM requires simple or complicated processing procedures, based on clinical requirements. Nevertheless, investigating the processing mechanisms of HM is challenging, leading to a lack of processing standardizations. A pivotal strategy to address the issue involves the analysis of dynamic transformation of chemical components between raw and processed products (Wu et al., 2018). MSI is particularly advantageous for this application due to its requirement for minimal sample preparation, thereby preserving the natural state of compounds. In HMs, although alkaloids serve as pharmacologically active constituents in numerous herbs, excessive concentrations of these compounds can lead to adverse toxic effects. Therefore, it is crucial to maintain alkaloid levels within an optimal range through adequate processing, thus ensuring efficacy and safe clinical use. MSI has been widely applied for *in situ* detection of alkaloid dynamic changes in HM processing such as Tibetan medicine Radix Aconiti (Tan et al., 2023), Fuzi (Wu et al., 2007a; Liu Y. et al., 2022), and Strychnos nux-vomica seed (Wu et al., 2007b). For example, Fuzi, the lateral root of *Aconitum carmichaelii* Debeaux (Family: Ranunculaceae), is a highly toxic HM. The processing mechanism behind detoxification of Fuzi has been uncovered using DESI–MSI combined with metabolomics analysis. The results demonstrated significant differences in the spatial distribution of six alkaloids among samples exposed to different steaming time points (Figure 2C). Among them, 4-h steaming was found to be the optimal steaming time to attenuate toxicity and preserve efficacy. In addition, 42 metabolic markers were identified to discriminate between raw Fuzi and those steamed for 4 h and 8 h (Lei et al., 2021). The chemical transformations, primarily involving the hydrolysis of diester-diterpenoid alkaloids, were visually depicted through DESI–MSI. Furthermore, MSI also serves as a valuable tool for evaluating the disparities in the chemical transformation that arise from different processing methods. Radix Aconiti, also known as Tie-bang-chui (TBC), is a perennial herb of the genus Aconitum pendulum Busch. and A. flavum Hand.-Mazz. dry roots and is a typical Tibetan medicine with remarkable efficacy and high toxicity (Wang et al., 2016). The Tibetan medicine is often processed by non-heating highland barley wine ((HBW) and fructus chebulae soup (FCS) to reduce toxicity. In another study, researchers used high performance thin-layer chromatography (HPTLC) and DESI-MSI to intuitively uncover the alkaloid change of TBC processed by FCS (F-TBC) and HBW (H-TBC). The finding indicated that the content of all six alkaloids decreased in F-TBC, while all five alkaloids decreased except aconitine increased in H-TBC. Further analysis revealed that the difference may be associated with a high concentration of acidic tannins in FCS. These chemicals can accelerate the hydrolysis of toxic diester alkaloids and complex with alkaloids to form insoluble substances, while H-TBC does not have this rule (Tan et al., 2023). Both crude and processed *P. notoginseng* exhibit high medicinal values due to their distinctive pharmacologic activities. MALDI–MSI was performed to visualize the dynamic changes among diverse metabolites during steaming. Dencichine, a hemostatic substance, gradually reduced with steaming time, verifying the superior hemostatic effects of raw *P. notoginseng* over steamed products. Alternatively, an increase in essential amino acids like arginine and glutamine was observed after steaming, accounting for the augmented blood-nourishing effects of the steamed form (Sun C. et al., 2021). The varying effects between raw and steamed *P. notoginseng* can also be attributed to the transformation of ginsenosides that occurs during the processing. Untargeted metabolomics identified 19 processing-associated markers. MALDI–MSI indicated that the major ginsenosides M-Rb1, R1, Rg1, Rb1, Rd, and Re were rich in the xylem and gradually decreased during processing (Fan et al., 2022). The transformation pathway of ginsenosides during processing was associated with chemical reactions such as deglycosylation, dehydration, hydration, acetylation, and isomerization. These studies can provide a clue for the distinct bioactivities between raw and processed *P. notoginseng*. Furthermore, an integral strategy combining multi-component characterization, non-target metabolomics, and MSI was proposed for quality control of processed Ligustri Lucidi Fructus [Oleaceae, mature fruit of *Ligustrum lucidum* Ait.] with different steaming times (0–12 h). MSI was employed to visualize four major processing-associated markers.

The comprehensive method, as opposed to conventional approaches, provides more compelling data for investigating HM processing mechanisms from a macroscopic perspective (Li et al., 2020). Likewise, MSI has been extensively utilized to identify chemical alterations between various raw and processed products, including raw and steamed Gastrodiae Rhizoma (Harkin et al., 2022), raw and steamed Aconiti Radix Cocta (Dai et al., 2022), raw and steamed Morindae Officinalis Radix (Qiao et al., 2022), raw and multi-steamed Panax quinquefolius (Li H. Z. et al., 2023). From a chemical standpoint, MSI has emerged as an efficient, rapid tool to unravel the processing mechanisms of HMs through the characterization of chemical structures, relative contents, and spatial distributions of specific metabolites during processing. This approach offers significant advantages for quality evaluation and control of HM processing, as well as for further standardizing processing methodologies. In summary, MSI establishes a direct link between the location of secondary metabolites and their physiological functions, metabolic differences across various conditions, and chemical transformations during processing. This information can guide targeted extraction of active metabolites and further optimize processing techniques.

## 4 MSI to elucidate HM mechanisms of action

The HM therapeutic effects are intricate and involve multiple components and target organs. This complexity poses a significant challenge for researchers in interpreting their pharmacologic actions. MSI has been employed to visualize the spatial distribution of active components as well as the metabolic status of endogenous metabolites in specific animal tissues. MSI-based metabolomics can offer valuable insights into the spatial alterations of endogenous biomarkers within tissues following drug administration, enabling a comprehensive understanding of the molecular-level pharmacological and toxic mechanisms of HMs (He et al., 2015).

### 4.1 Animal tissue distributions of active ingredients

The majority of HM compounds exhibit heterogeneous distribution *in vivo*, specifically targeting specific organ regions to achieve curative efficacies (Karlsson and Hanrieder, 2017). Their heterogeneous distributions in pathological tissues can provide valuable information for drug development. However, excessive accumulation in target organs can cause toxic side effects (Jiang et al., 2021). Hence, monitoring the spatial distribution and abundance of these compounds and their metabolites is a prerequisite for exploring their pharmacokinetic and pharmacodynamic properties. In addition to medicinal plant tissues, MSI is an efficient method for assessing the spatial dynamics of active constituents in animal tissues.

For example, tetrandrine, derived from the Chinese herb *Stephania tetrandra* S. Moore, is a notable natural product with remarkable anti-tumor bioactivity (Yu et al., 2018). MALDI-MSI result indicated that tetrandrine was widely distributed in the lung basal region, kidney cortex, and heart apex region. Despite homogeneous distribution in the liver, the elimination rate of tetrandrine was slow (Tang et al., 2019). Excessive accumulation of tetrandrine in these organs can trigger potentially pulmonary (Jin et al., 2011), hepatic (Wang et al., 2011), and renal (Qi et al., 2013) toxicities. *Uncaria* species (Rubiaceae) are used to treat central nervous system (CNS) disorders. Monoterpene indole alkaloids serve as the main active substances. Gao et al. successfully adopted DESI–MSI to locate and quantify seven *Uncaria* alkaloids in 13 brain regions. These alkaloids exhibited a heterogeneous distribution pattern across brain regions. Of note, 5 min post-administration, the pineal gland, a brain region that regulates biological rhythms, contained a higher abundance of alkaloids compared with other brain regions (Gao et al., 2022). Their enrichment phenomenon in specific region had an instructive significance in future pharmacodynamic studies. Ginsenoside Rg1 is a main active compound in the family Araliaceae, including *P. notoginseng* and Panaxginseng. Its distributions in heart, liver, spleen, lung, brain, and kidney tissues were revealed by nano-DESI combined with LC–MS/MS (Wei et al., 2021). The compound was mainly concentrated in the kidney pelvis, while there were no obvious accumulation trends in other organs. Rg1 also appeared in the CNS pons and medulla oblongata 15 min after intravenous administration. These cumulative findings provide new directions for further discovery of Rg1 effects. The spatial distribution of HM components within tissues serves as an informative indicator for predicting their bioactivities and identifying their target organs.

### 4.2 Pharmacologic and toxicologic mechanisms

Beyond compound distribution, MSI enables the visualization of the spatial positioning and dynamic alterations of endogenous metabolites within specific animal tissues. Recently, MSI-based spatial metabolomics has emerged as a novel way to visualize efficacy-related biomarkers and associate spatial distributions with histopathological staining. These approaches are more beneficial for detailing the pharmacological and toxicological mechanisms of HM from a spatial view compared with traditional metabolomics.

For example, pterostilbene, a polyphenolic compound extracted from grapes and blueberries (Dutta et al., 2023), is also the primary active substance in *Resina Draconis*. Ban et al. (2024) used DESI–MSI-based spatial metabolomics to probe the intricate mechanisms of pterostilbene in cerebral ischemia/reperfusion injury. Pterostilbene was widely distributed across the ischemic cortex lesion site. The specific distribution patterns of pterostilbene in ischemic brain tissue played a crucial role in explaining its unique pharmacological characteristics. Furthermore, the *in situ* reversal of the spatial distribution and abundance of altered metabolites, including glucose, glutamate, and creatine, in rat brain tissues, contributes to a deeper understanding of the underlying mechanisms of pterostilbene.

In a representative study regarding a TCM formula, XueFuZhuYu decoction, spatial metabolomics based on nano-DESI was employed to unveil the pathological progression and therapeutic effectiveness in the treatment of traumatic brain injury (TBI). According to ion imaging, phosphatidylcholines, lysophosphatidylcholine, and diacylglycerols were highly increased in the midbrain and thalamus after treatment. The accumulation was associated with the activation of “self-repair” mechanisms in the lesion area, activated by neuroinflammation during the chronic TBI phase. A total of 10 significant metabolic pathways were impacted following TBI treatment, and six related formula target proteins were identified, shedding light on the molecular mechanisms of XueFuZhuYu decoction in TBI treatment (Li Y. et al., 2021). Similarly, the protective mechanism of Shuangshen Ningxin Capsule against myocardial ischemia has been evaluated by spatial metabolomics (Lian et al., 2024). Researchers have applied DESI–MSI to search for brain biomarkers reflecting the warm and cool properties of ginseng and American ginseng, to explain the modern scientific connotation of the medicinal properties of HM (Huang et al., 2023). Therefore, MSI-based spatial metabolomics could become a reference methodology for interpreting TCM theory. In addition to pharmacological mechanisms, it is crucial to comprehensively describe the toxicological processes of candidates for drug discovery and development (Spruill et al., 2022). MSI can also serve as a powerful tool for investigating the toxicological mechanisms of HM, such as nephrotoxicity associated with aristolochic acids (Wang et al., 2020) and hepatotoxicity induced by Heshouwu [Polygonaceae; Root tubers of *Polygonum multiflorum* Thunb] (Jiang H. Y. et al., 2022). In conclusion, the integration of MSI-based spatial metabolomics with biochemical and histopathological examination can further deepen our understanding of the pharmacological and toxicological mechanisms of HM.

## 5 Future perspectives and challenges

Over the decades, MSI has benefited from continuous improvements in ion sources and mass analyzers. Various commercial ionization techniques, with higher spatial resolution and greater sensitivity, have been developed to accommodate the analysis of various metabolites. In the MSI process, abundant information on molecular structures, original spatial location, and relative contents of molecules are simultaneously provided without complex sample pretreatment. With its increasing popularity and technological superiority, MSI is expected to become a major tool in HM. Additionally, researchers should strive to expand MSI for other HM applications. For example, MSI might soon serve as a powerful tool for predicting HM component bioactivities, investigating unknown compounds according to their spatial distribution patterns, detecting HM pesticide residues, or authenticating HM combining with PCA and OPLS-DA. In terms of the therapeutic mechanisms of HM, most research has been focused on the evaluation of HM efficacy against brain disorders. Given its characteristics of *in situ* analysis and high spatial resolution, MSI will assist in unlocking dynamic metabolic processes in the tumor or disease microenvironment. Despite the unique strengths and novelty of MSI compared with other analytics, several major intrinsic limitations and challenges remain to be addressed. First, vast numbers of isomeric compounds in HM cannot be accurately distinguished by MSI alone due to its inability to perform chromatographic separation. Therefore, ion images obtained may contain a sum of substantial isomers. Complementary techniques are required to overcome the limitation. Although the introduction of tandem MSI can partially address this problem (Bednařík et al., 2022), distinguishing polysaccharides and isomers remains challenging. The integration of IM separation has enabled more isomer discrimination by determining the collision cross-section of metabolites based on shape, size, and ion charge (Jiang L. X. et al., 2023). Besides, it is also essential to use specific chemical reactions or structure-specific derivatization to identify isomers (Zhan et al., 2021). Second, accurate quantitation of HM ingredients and their metabolites between different regions of the same tissue or between different tissues using MSI techniques is a prerequisite for exploring their pharmacokinetic-pharmacodynamic properties. However, absolute quantitation remains a considerable challenge owing to the impacts of matrix effects and ion suppression on sample pixels during ionization (Alexandrov, 2023). Numerous efforts have been reported to achieve the relative or absolute MSI analysis. For example, the compensation for matrix effects is necessary for accurate quantification in MSI. As previously outlined (Unsihuay et al., 2021), various normalization strategies, appropriate sample preparation, and calibration measures have garnered significant attention for their potential to mitigate matrix signal interference. Third, enhanced confidence in compound/metabolite identification is in high demand. Generally, coupling MSI with tandem MS has proven to be an effective approach for acquiring additional structural information, thereby facilitating more reliable metabolite identification (Yin et al., 2023). Fourth, the high dimensionality of MSI data poses challenges in data analysis (e.g., data handling, processing, integration, storage). Consequently, specialized software and algorithm optimization are necessary to effectively manage and analyze the large volume of data generated in MSI experiments. Multiple vendors offer most of the currently used software, such as SCiLS™ Lab and MetaboScape^®^ software from Bruker, ImageQuest from Thermo, High Definition Imaging Software from Waters Corp, and so on. In addition, some comprehensive software provided by Thermo Fisher Scientific, Agilent Technologies, SCIEX, etc. also have the function of processing MSI data, including PeakView^®^, MassHunter, and XCMS. Moreover, lipostarMSI is a newly developed and comprehensive software capable of executing all steps in the processing flow of MSI data. Per E. Andrén’s team has developed a space-efficient, direct access data compression algorithm for MSI to achieve efficient data archiving (Källback et al., 2018). Subsequently, an innovative spectral cross-normalization algorithm was introduced, incorporating intensity profile normalization and peptide mass resampling, along with a secondary normalization step. This approach adeptly minimizes technical discrepancies within the data, while preserving the integrity of the underlying biological information (Boskamp et al., 2021). Moreover, the establishment of public repositories and databases for published and shareable MSI data, coupled with the application of data mining tools/software, will facilitate convenient access to MSI techniques and promote its wider adoption. In the experiment, the MSI technique is capable of simultaneously detecting thousands of metabolites, which might lead to poor molecular specificity. Therefore, specific scanning modes such as Single Ion Monitoring need to be established during MS data collection to target the drugs and their metabolites of interest. Compared with LC-MS data, MSI also confronts the challenge of lower detection limits. Although some studies have been conducted to measure the absorption and distribution of HM components *in vivo* by increasing the medication dose, it may give rise to the potential risk of stress response and metabolic homeostasis disruption during high-dose administration. The advancements in mass analyzers in recent years have contributed to solving the problem. For example, in addition to the most widely used TOF, the introduction of Fourier transform ion cyclotron resonance and orbitrap mass analyzer is characterized by high mass resolving power and mass accuracy, significantly enhancing the detection limits and molecular specificity for MSI analysis (Kihara et al., 2017). Lastly, metabolites differ among preferred ion sources (Alexandrov, 2023). A single MSI technique cannot simultaneously detect all analytes of interest. Therefore, selecting an appropriate ionization approach, tailored to the characteristics of the target compound, is crucial for practical applications.

## 6 Conclusion

MSI plays an increasingly pivotal role in HM research, particularly in the analysis of spatial information about molecules within heterogeneous tissues. This technique is crucial for unveiling the biosynthetic pathways of active ingredients, providing insights into processing mechanisms, and understanding the target organs and actions of HMs by visualizing compound spatial distribution. This review provides a comprehensive overview of the three most common MSI techniques, highlighting their current applications in HM and strategies for improving the technique. It is anticipated that advancements in spatial resolution, matrix selection, and sample preparation will further enhance the discovery of novel HM compounds. With its exceptional performance and accessibility, MSI holds immense potential to revolutionize our understanding of HM, paving the way for more effective drug development and therapeutic strategies.
